# Comparative Functional Responses Predict the Invasiveness and Ecological Impacts of Alien Herbivorous Snails

**DOI:** 10.1371/journal.pone.0147017

**Published:** 2016-01-15

**Authors:** Meng Xu, Xidong Mu, Jaimie T. A. Dick, Miao Fang, Dangen Gu, Du Luo, Jiaen Zhang, Jianren Luo, Yinchang Hu

**Affiliations:** 1 Pearl River Fisheries Research Institute, Chinese Academy of Fishery Sciences, Key Laboratory of Tropical and Subtropical Fishery Resource Application and Cultivation, Ministry of Agriculture, Guangzhou, China; 2 Institute for Global Food Security, School of Biological Sciences, Queen’s University Belfast, MBC, 97 Lisburn Road, Belfast, BT9 7BL, Northern Ireland, United Kingdom; 3 College of Fisheries and Life Science, Shanghai Ocean University, Shanghai, China; 4 Department of Ecology, College of Agriculture, South China Agricultural University, Key Laboratory of Agro-environments in Tropics, Ministry of Agriculture, Guangzhou, China; National University of Singapore, SINGAPORE

## Abstract

Understanding determinants of the invasiveness and ecological impacts of alien species is amongst the most sought-after and urgent research questions in ecology. Several studies have shown the value of comparing the functional responses (FRs) of alien and native predators towards native prey, however, the technique is under-explored with herbivorous alien species and as a predictor of invasiveness as distinct from ecological impact. Here, in China, we conducted a mesocosm experiment to compare the FRs among three herbivorous snail species: the golden apple snail, *Pomacea canaliculata*, a highly invasive and high impact alien listed in “100 of the World's Worst Invasive Alien Species”; *Planorbarius corneus*, a non-invasive, low impact alien; and the Chinese native snail, *Bellamya aeruginosa*, when feeding on four locally occurring plant species. Further, by using a numerical response equation, we modelled the population dynamics of the snail consumers. For standard FR parameters, we found that the invasive and damaging alien snail had the highest “attack rates” *a*, shortest “handling times” *h* and also the highest estimated maximum feeding rates, 1/*hT*, whereas the native species had the lowest attack rates, longest handling times and lowest maximum feeding rates. The non-invasive, low impact alien species had consistently intermediate FR parameters. The invasive alien species had higher population growth potential than the native snail species, whilst that of the non-invasive alien species was intermediate. Thus, while the comparative FR approach has been proposed as a reliable method for predicting the ecological impacts of invasive predators, our results further suggest that comparative FRs could extend to predict the invasiveness and ecological impacts of alien herbivores and should be explored in other taxa and trophic groups to determine the general utility of the approach.

## Introduction

Attempts to understand determinants of the invasiveness and ecological impacts of alien species have led to various comparisons of species traits amongst invaders and natives [1]. This has yielded mixed results at best, with no single traditional species trait emerging as a useful predictor of invasiveness or ecological impact [2,3], where “invasiveness” is the rate of establishment and/or spread and “impact” is the effect on native species populations [4]. While invasion history is a good predictor of invader impact [5], we ideally need measurable traits of species that can predict existing, emerging and future invasiveness and ecological impact [3,6]. There has been recognition that high impact invasive species are better able to capitalize on resource availability than native species and non-invasive alien species [7,8]. However, a general approach that relates the consumer-resource interaction to invasiveness and impact across taxonomic and trophic groups has been lacking [3]. Consumer-resource interactions have long been the focus of ecology, and the “functional response” (FR), describing the relationship between resource density and resource consumption rate, has been recognized as a crucial process influencing the dynamics of populations and communities and the stability of food webs [9–12]. Three types of FRs, at least, have been identified [3,13], with Type II and III most common and of most interest in comparative FR studies [3]. In the Type II FR, the amount of resource consumed approaches an asymptote hyperbolically as resource density/availability increases; this corresponds to declining proportional consumption. This represents inverse density dependence, where resources at low density are more likely to be consumed, thus potentially leading to de-stabilization of the resource population. The Type III FR is characterized by an asymptotical sigmoidal curve as resource density/availability increases; this corresponds to an increase and then decrease in proportional consumption and as such may impart a refuge when resource density falls below a threshold level, potentially stabilizing consumer-resource dynamics [3].

More recently, deriving FRs of invasive and native species has been proposed as a promising species trait comparison that closely associates with the ecological impacts of invasive species [3,6,14]. Through comparisons of invader and native FR magnitudes (i.e. asymptote) and Type (II and/or III), the likely population-level consequences for native prey can be examined. Several empirical studies have adopted this approach, and shown that higher FRs than natives predicted actual invader impacts in the field [6,15–17]. As detailed by Dick et al. [3], this method can be expanded to test the impact of two or more invaders, two or more populations of the same invader, and the biotic and abiotic context-dependencies of invader impacts.

The comparative functional response approach has, however, thus far only been explored with respect to invader and native predator-prey relationships, but not yet with respect to the invasiveness and ecological impacts of herbivorous aliens. While FRs are most readily understood and measured as a predator-prey relationship, there is no reason that FRs cannot be measured for any consumer of a resource [3]; thus, here, we attempt to extend the method to an animal-plant interaction using herbivorous snails as a study system. Further, as invasiveness and ecological impact are separate aspects of aliens species dynamics, and indeed do not predict one another [4], we also explore in the present study the utility of comparative FRs of invaders with respect to different invasiveness, in addition to understanding and predicting ecological impacts of invaders.

Therefore, in this study, we firstly conducted a mesocosm experiment to determine the FRs of three herbivorous snails: the highly invasive and ecologically damaging alien “golden apple snail” (*Pomacea canaliculata*, Lamarck 1822); the non-invasive and low impact alien “great ramshorn snail” (*Planorbarius corneus*, Linnaeus, 1758); and a trophically analogous and ecologically benign native (*Bellamya aeruginosa*, (Reeve) Yen, 1943), when feeding on four common aquatic plants. We then associated the FRs with the invasiveness and ecological impacts of these snails to explore whether, as predicted from the theory [3], the invasive alien species had different standard FR parameters and higher FR magnitudes than the non-invasive alien and the native species, and whether the non-invasive species had intermediate FR parameters. Then, we used a numerical response equation to explore the population development of snails with different FRs. We conclude with an assessment as to the utility of the comparative FR method to understand and predict the invasiveness and ecological impacts of alien species across taxonomic and trophic groups.

## Materials and Methods

This work was conducted according to relevant national and international guidelines. The target species, *Pomacea canaliculata*, is listed in “100 of the World's Worst Invasive Alien Species” and has caused great damage to agriculture and the economy. In our study, we conducted a manipulative experiment in an outdoor shade-house to test plant consumption by this species and this did not involve anesthesia, euthanasia or any kind of animal sacrifice.

### Experimental organisms

The golden apple snail (*Pomacea canaliculata*; Gastropoda, Ampullariidae) is native to freshwater wetlands of South America [18] and has been introduced widely into Asia since the 1980s [19]. This snail is known as a notorious invasive species, exerting significant detrimental influences on agricultural production [18], wetland plants [20], and ecosystem functioning [21]. Further, this species is the main intermediate host of *Angiostrongylus cantonensis* that can cause a potentially fatal human disease, eosinophilic meningitis [22]. *Pomacea canaliculata* has been listed in “100 of the World's Worst Invasive Alien Species” by the International Union for Conservation of Nature (IUCN) [23]. This species has separate sexes that can be morphologically distinguished by the curve of the operculum. Females lay pink batches of 25–500 eggs and newborns can reach maturity within 60–85 days [24]. The maximum height and weight of this species, according to our field surveys, can reach 7.5 cm and 66 g respectively. Our previous study has also shown that *Po*. *maculata*, a highly similar species to *Po*. *canaliculata*, did not occur in our research region, ensuring that we were using the invasive *Po*. *canaliculata* in the present study [25]. The great ramshorn snail (*Planorbarius corneus*; Gastropoda, Pulmonata, Planorbidae) is native to European freshwaters [26–28] and widely introduced to China as an Ornamental species [29]. It inhabits mainly central European wetlands and temporary basins with a standing body of water and aquatic plants [27,30]. It is gonochoristic and oviparous, and the adults may be up to 2.5 cm in diameter and about 1.5 g in body weight [29]. To date, there are no accurate records about where and when *Pl*. *corneus* was introduced into China. However, since it was imported as an aquarium species, we inferred that it was likely introduced into South China in the last decade, given that South China (particularly Guangzhou City) is the national center of the Chinese aquarium trade. *Planorbarius corneus* is found occasionally in the field according to our field surveys in Guangdong province over the last five years and communications with local people in rural areas of Guangzhou. To date, *Pl*. *corneus* has not been reported to cause any ecological damage in China, however, as this species has increasingly become a common alien species in aquaria, it is necessary to assess its potential invasiveness and ecological impact. The Chinese native snail (*Bellamya aeruginosa*; Gastropoda, Prosobranchia, Valvatidae) is common and widespread in China. This species is gonochoristic and reproduction ovoviviparous, with maximum adult size around 2–3 cm. When shell lengths reach 1.5 cm (0.8 g in body weight), females begin to reproduce and the reproductive cycle is about 6 months; the average number of newborns in a year is about 50 individuals [31]. *Bellamya aeruginosa*, as a native species, has not been reported to cause any ecological/agricultural problems, but some research suggests that it could also be an intermediate host of *Angiostrongylus cantonensis* [32]. These three snail species were chosen as the test species because they are all herbivorous, with overlapping dietary requirements [29,33,34], the invasive *Po*. *canaliculata* is one of the most notorious alien species, while *Pl*. *corneus* is an emerging invader and *B*. *aeruginosa* is the most common and ecologically similar native snail.

All three snail species were cultured in the ponds of the Peal River Fisheries Research Institute (23°04′9.42″ N; 113°12′52.68″ E) in Guangzhou City, China, in 2014. Each species was fed with *Najas guadalupensis* (one of the most common aquatic weeds used in aquaria) and housed separately in ponds (5 m × 4 m × 1.5 m) with re-circulating water systems. Prior to the experiment, the three snail species were held without food for 24 h to allow for standardization of hunger levels, a standard method in comparative FRs [6]. Snails with similar body size were deliberately used in the experiment to control for the possible confounding effect on FRs of body mass (the mean body mass ± SE was as follows: *Po*. *canaliculata* 1.15 ± 0.02 g, *Pl*. *corneus* 1.10 ± 0.02 g and *B*. *aeruginosa* 1.17 ± 0.02 g; there was no significant difference in body mass, one-way ANOVA, *F*_2, 249_ = 2.89, *P* = 0.06). Four species of aquatic vegetation (water spinach: *Ipomoea aquatica*; washington grass: *Cabomba caroliniana*; water wisteria: *Hygrophila difformis*; and Indian toothcup: *Rotala indica*) were chosen as food resources as they are common in freshwater ecosystems and were readily fed upon by the three snail species in our pilot observations. These four plants were collected one week prior to the experiments, and were individually cultivated in 25 L holding tanks.

### Mesocosm experimental design

Comparative functional response (FR) experiments were conducted in an out-door shade-house (25 m × 15 m × 5 m) in 2014. The experiment started at 08:00 on July 15 and ended at 08:00 on July 17, with the natural temperature range over the experimental duration being 26–29°C at night-time (approximately 12 h) and 29–35°C during daytime. A three-way split-plot design was used; the first factor was the species of aquatic plant (*I*. *aquatica*, *C*. *caroliniana*, *H*. *difformis* and *R*. *indica*), with each species added separately to 7 25-L holding tanks (45 cm × 35 cm × 25 cm), that is, 7 holding tanks for each of the 4 plant species (= 28 holding tanks). Each holding tank was treated with one of seven plant densities as wet mass at 0.5, 1, 2, 4, 6, 8, 10 g and density used as the second factor. Within each holding tank, the plants were allocated to 4 net cages (15 cm × 15 cm × 15 cm), with 3 of the net cages supplied with one individual of each of the three species of snail, while the remaining net cage without any snail was used as a control. The species of snail (including control) was used as the third factor. These 28 holding tanks constituted one block, and in this experiment three blocks were replicated simultaneously. The whole experiment involving the 84 holding tanks was run for 48 h, after which the wet mass of the aquatic plant species was determined. Firstly, the potential plant growth during the experiment was determined through subtracting initial plant biomass from the biomass in control net cages after 48 hours (M_control 48 hours_—M_control time zero_). The consumption by snails was then determined by adding net mass consumption (M_treatment time zero_—M_treatment 48 hours_) to the potential plant growth. A three-way split-plot ANOVA was used to examine the mean plant mass consumption with respect to species of snail, species of plant and initial plant biomass (= density in an FR experiment), and the “blocks/plant species/initial biomass/snail species” was used as error structure [35].

### Functional response analyses

We firstly determine what Types of FR (II or III) were displayed. The proportion of plant consumed by snails as the polynomial function of plant biomass was modelled. A significant negative first order term indicates a Type II FR, while a significant positive first order term, followed by a significant negative second order term, represents a Type III FR [13]. We then derived the FRs for each of the three snail species towards each of the four plant species. Rogers’ random predator equation is commonly used to describe Type II FRs where the resource is not replaced as it is consumed; we thus used Rogers’ random equation as we did not replace the plant biomass consumed by snails [36–38]:

Ne=N(1−exp(a(Neh−T))) (1)

where *Ne* is the biomass of plant, *N* is the initial biomass, *a* is the “attack rate”, *h* is the “handling time” and *T* is the total experiment time. This recursive function can be resolved using the *Lambert W* function [39]: ,

$$Ne=N-\frac{W(ahN\;\text{exp}(-a(T-hN)))}{ah}$$ (2)

The parameters *a* and *h* for the FR of each snail species feeding on each plant species were estimated using non-linear least squares regression and the *lambert W* function of the package “emdbook” [40]. The estimated maximum feeding rate was calculated as 1/*hT* where *T* is the experiment duration. The parameters of *a* and *h* in predator-prey systems are classically interpreted as the “attack rate” and “handling time” respectively, but can also include other elements of consumption, such as digestion rate [11]. In the context of herbivores, attack rates may translate as ingestion rates (the product of bite frequency and bite volume) and handling times as chewing time [41–43]. However, here we do not interpret the parameters mechanistically, rather, we use them phenomenologically and for comparative purposes [44]. FR data for each snail species on each plant species were firstly bootstrapped (n = 10 each) and FR parameters compared among each snail-plant combination. Then, we calculated the mean feeding parameters for each snail species by bootstrapping (n = 30 each) the FRs with respect to all the four plant species combined [44]. The comparison of parameters was achieved by ANOVA and Tukey’s *post hoc* tests.

To further differentiate and visualize the FRs of the three snail species across all plant species combined, we constructed and compared the 95% confidence intervals around the mean FR curves of each snail species (see [38]). Raw consumption data for each snail species toward the four plant species combined were bootstrapped (n = 999), subsequently applying Rogers’ random predator equation to each data set to construct 95% confidence intervals around the mean functional response curves; the starting values of *a* and *h* for each bootstrapped data set were the same as those derived from the original data for each snail species [38].

### Simulation of snail population dynamics

For prediction of the feedback effect of FRs on the snails themselves, we used the Rosenzweig-MacArthur model framework to simulate the plant and snail biomass dynamics [45,46]. Although this model was initially used to describe predator-prey dynamics, it has been successfully applied to predict the interaction between herbivores and plant resources [47]. The population growth model (numerical response) of the herbivorous snails is as follow:

$$\frac{dy}{dt}=fy(N-\frac{W(ahN\;\text{exp}(-a(T-hN)))}{ah})-\mu y^{2}$$ (3)

where *f* is the efficiency of converting food into biomass and *μ* is *per capita* loss rate with increasing biomass of snails. In this study, we used biomass rather than numbers of snails to represent the population growth; although the increasing number of snails represent the real numerical response, this would need to include more parameters, such as reproductive characteristics, survival rate of juvenile and adult snails, and generation times. Given that these parameters are not available now for all three snails, we instead elected to use the biomass growth involving conversion efficiency and loss rate to characterize and compare the general population growth potentials among the three snail species. We assumed that the loss rate of snails increased with biomass (i.e. *μy*) rather than a fixed value, so loss amount was μy*y. The other parameters are the same as those in Eq (2). The dependent effect *μ* has been investigated for the invasive *Po*. *canaliculata*, and the value is about 0.01 [48]. The conversion efficiency *f* of snails was about 0.05 and it was also already found to be similar for the alien and native Ampullariidae in the USA [34]. However, considering the possible lower conversion efficiency *f* and higher dependent effect *μ* for the alien species that encounters a novel environment and consumes novel plant resources, we varied both parameters when conducting this simulation. We firstly fixed the *μ* as 0.01 and changed the *f* from 0.01 to 0.09, then we fixed the *f* as 0.05 and changed the *μ* from 0.005 to 0.025 in the simulations.

All statistical analyses and simulations were performed in R, version 3.1.1 [49].

## Results

### Functional response experiment

**Fig 1:**
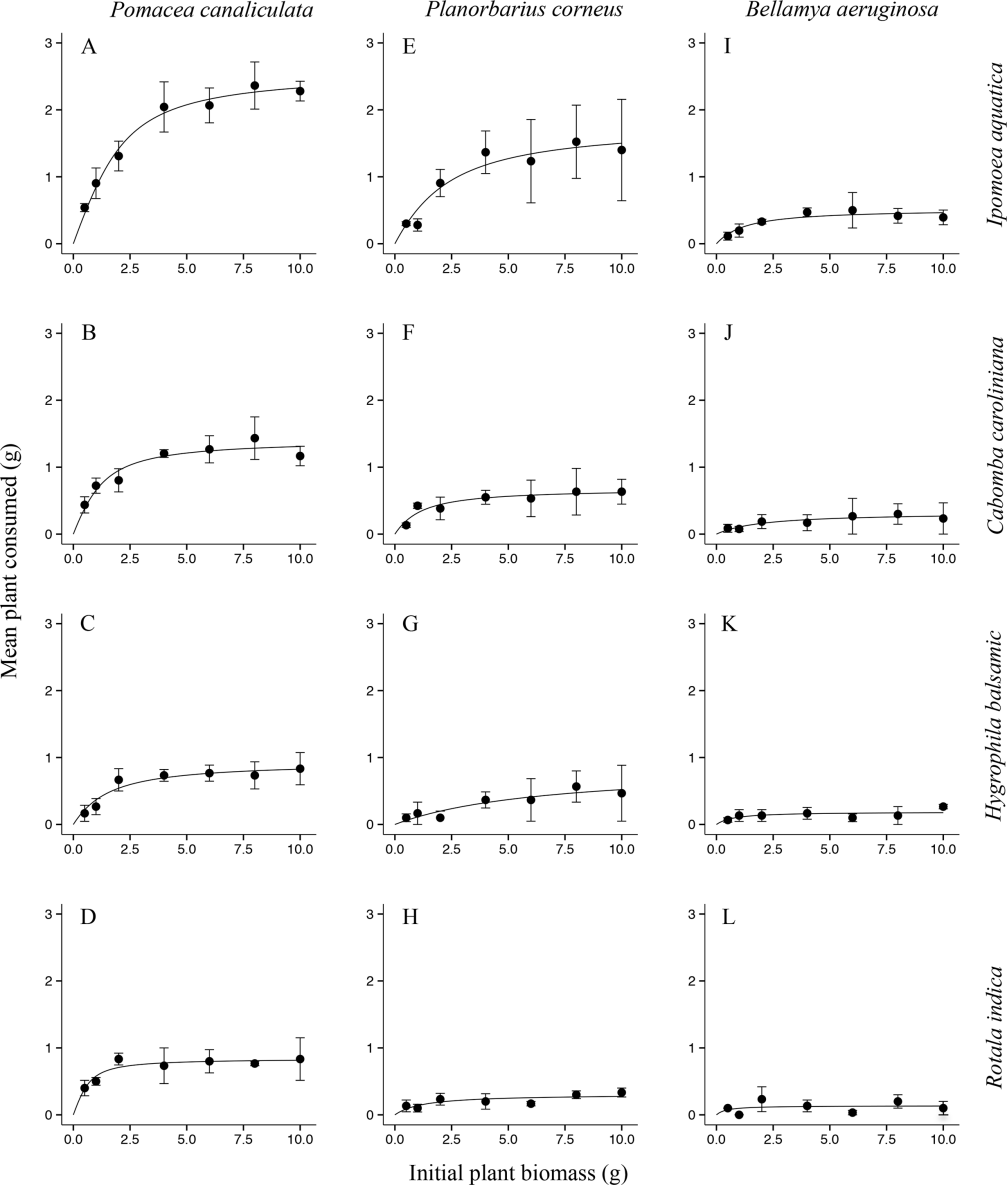
Functional response curves of the invasive and high impact alien, the non-invasive and low impact alien, and the benign native snail species, when feeding on four plant species. The invasive alien species *Pomacea canaliculata* fed on (A) *Ipomoea aquatica*, (B) *Cabomba caroliniana*, (C) *Hygrophila difformis* and (D) *Rotala indica*, and the non-invasive alien *Planorbarius corneus* fed on these same plant species (E, F, G, H) and similarly for the native *Bellamya aeruginosa* (I, J, K, L); modelled by the Rogers’ random predator equation for a Type II response; *n* = 3 per initial plant biomass. Error bars represent standard errors.

**Fig 2:**
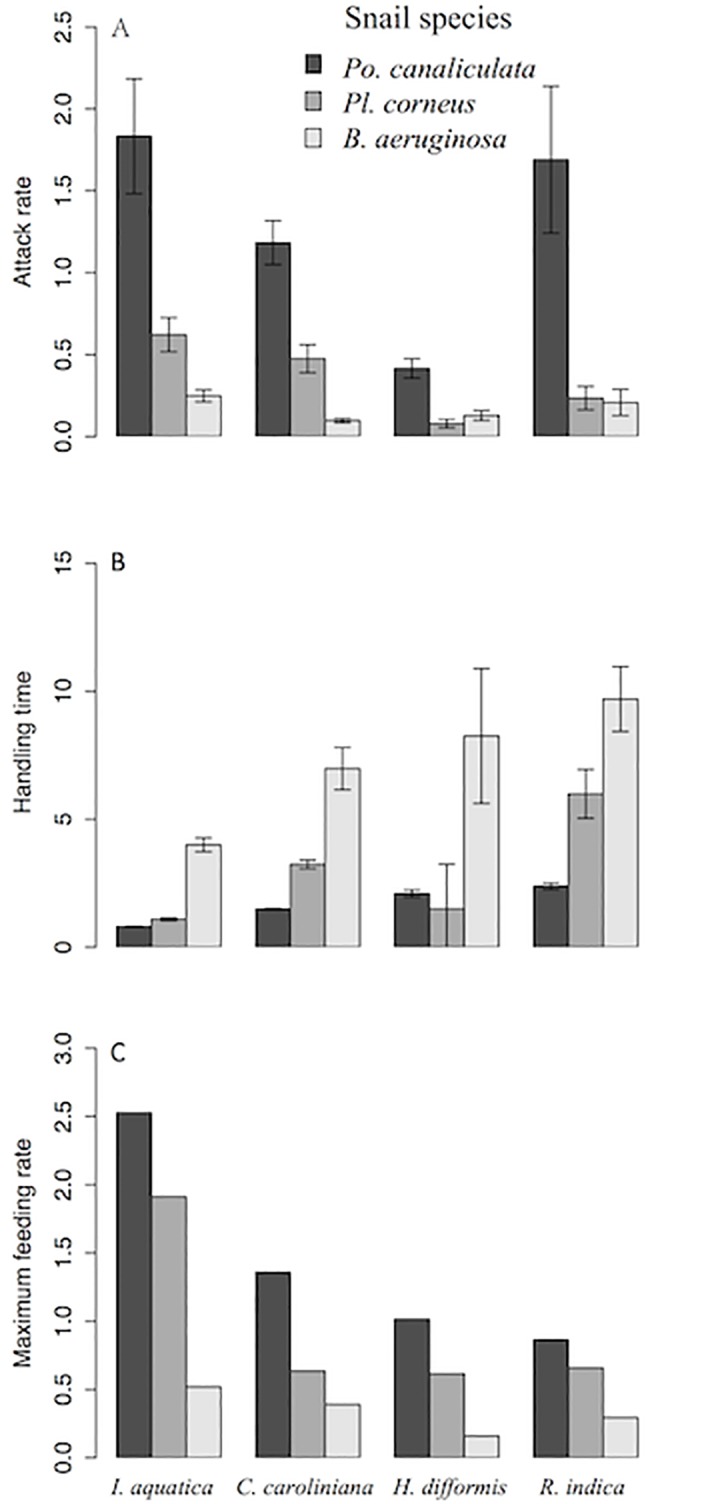
Comparison of functional response parameters associated with the three snail species feeding on four plant species. The mean (A) attack rate *a*, (B) handling time *h* and (C) maximum feeding rate *1/hT* were respectively derived from bootstrapping (n = 10 each) the parameter values of each of three snail species (*Pomacea canaliculata*, *Planorbarius corneus* and *Bellamya aeruginosa*) feeding on each of four plant species (*Ipomoea aquatica*, *Cabomba caroliniana*, *Hygrophila difformis* and *Rotala indica*). Error bars represent standard errors.

**Fig 3:**
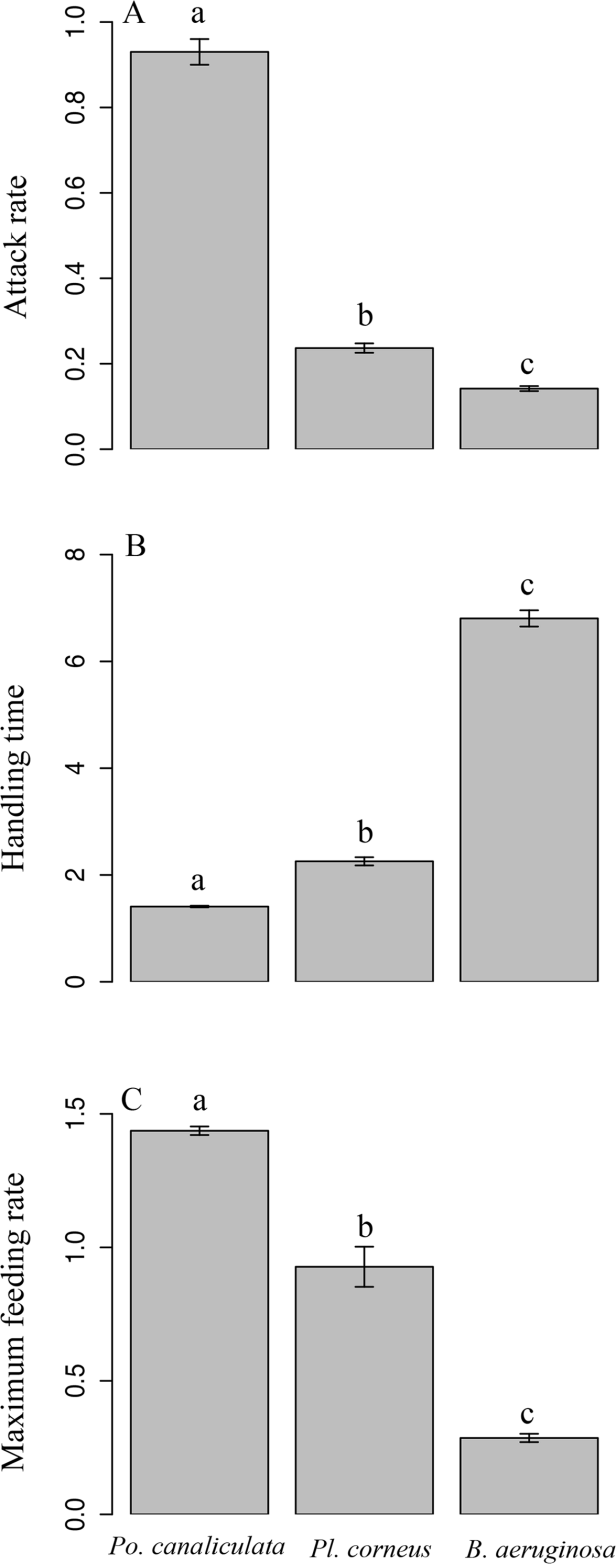
Comparison of mean functional response parameters associated with invasive alien, non-invasive alien and native snail species. The mean (A) attack rate *a*, (B) handling time *h* and (C) maximum feeding rate *1/hT* were respectively derived from bootstrapping (n = 30 each) the parameter values of each of three snail species (*Pomacea canaliculata*, *Planorbarius corneus* and *Bellamya aeruginosa*) feeding on all four plant species (*Ipomoea aquatica*, *Cabomba caroliniana*, *Hygrophila difformis* and *Rotala indica*). Different letters indicate significant differences (Tukey’s tests, *p* < 0.05). Error bars represent standard errors.

**Fig 4:**
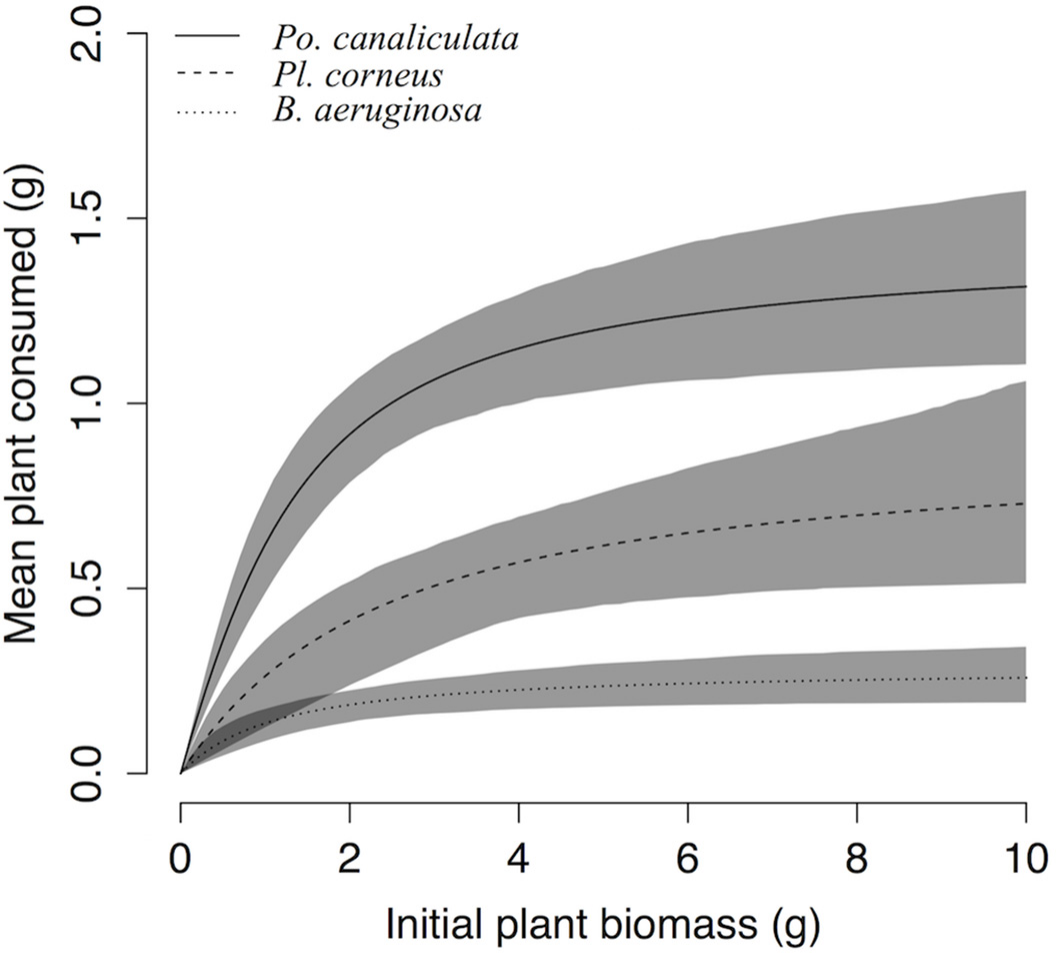
Functional response curves for the invasive alien, non-invasive alien and native snail species. The curves are the Rogers’ random predator equations fitted for each of three snail species (*Pomacea canaliculata*, *Planorbarius corneus* and *Bellamya aeruginosa*) when feeding on the four plant species combined (*Ipomoea aquatica*, *Cabomba caroliniana*, *Hygrophila difformis* and *Rotala indica*). Shaded areas are bootstrapped 95% confidence intervals (n = 999 bootstraps each).

Polynomial regression indicated that all three species of snail exhibited Type II FRs toward all four plant species (Fig 1), as revealed by the significantly negative first order terms (S1 Fig; S1 Table). Attack rates *a* of *Po*. *canaliculata* feeding on the four plant species were all significantly higher than those of both other snails (Tukey’s test, *P* < 0.001; Fig 2A), and those of *Pl*. *corneus* feeding on *I*. *aquatica* and *C*. *caroliniana* were intermediate, and when feeding on the other two plants those of *Pl*. *corneus* were not significantly different from those of *B*. *aeruginosa*. Handling times *h* of *Po*. *canaliculata* were all significantly lower than those of *B*. *aeruginosa* (Tukey’s test, *P* < 0.001; Fig 2B), and those of *Pl*. *corneus* were intermediate except when feeding on *H*. *difformis*. Estimated maximum feeding rates 1/*hT* of *Po*. *canaliculata* were all significantly higher than those of *B*. *aeruginosa* (Tukey’s test, *P* < 0.001; Fig 2C), and those of *Pl*. *corneus* were intermediate. Correspondingly, for each snail species over all plant species combined, mean attack rate *a* was significantly greater for invasive *Po*. *canaliculata* in comparison to both non-invasive *Pl*. *corneus* and native *B*. *aeruginosa* (Tukey’s test, *P* < 0.001 and *P* < 0.001; Fig 3A); mean handling time *h* was significantly lower for *Po*. *canaliculata* compared to both *Pl*. *corneus* and *B*. *aeruginosa* (Tukey’s test, *P* < 0.001 and *P* < 0.001; Fig 3B); and mean estimated maximum feeding rate 1/*hT* was significantly higher for *Po*. *canaliculata* compared to both *Pl*. *corneus* and *B*. *aeruginosa* (Tukey’s test, *P* < 0.001 and *P* < 0.001; Fig 3C). The mean attack rate, handling time and estimated maximum feeding rate for the non-invasive *Pl*. *corneus* was intermediate in all cases (Fig 3A, 3B and 3C). The invasive *Po*. *canaliculata* had the highest FR, the native *B*. *aeruginosa* had the lowest FR, and the non-invasive *Po*. *canaliculata* had an intermediate FR, as revealed by the little overlap of 95% confidence intervals among the three snail species across all four plant species combined (Fig 4).

### Modelling snail population dynamics

**Fig 5:**
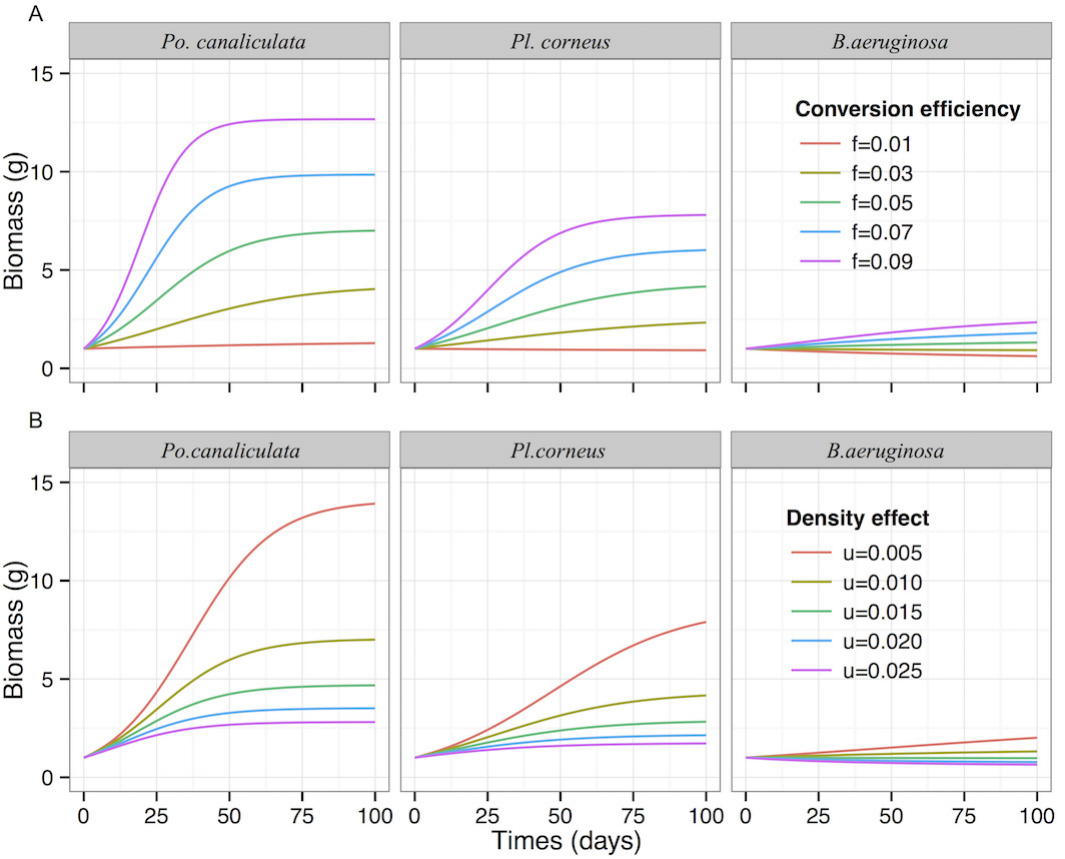
Simulated population growth of *Pomacea canaliculata*, *Planorbarius corneus* and *Bellamya aeruginosa*. (A) when three species have different conversion efficiency and (B) when three species have different *per capita* biomass loss rate. The functional response parameters are derived from the experiments and *a* = 0.93, *h* = 1.41 for *Pomacea canaliculata*; *a* = 0.24, *h* = 2.26 for *Planorbarius corneus*; *a* = 0.14, *h* = 6.80 for *Bellamya aeruginosa*.

Over time, when the three snails had the same conversion efficiency and *per capita* biomass loss rate, the biomass of *Po*. *canaliculata* increased most rapidly and reached the highest value, while for *Pl*. *corneus* and *B*. *aeruginosa* the biomass increased slowly and even decreased (Fig 5A and 5B). For *B*. *aeruginosa*, due to the low functional response, the varying conversion efficiency and *per capita* biomass loss, this species did not significantly change biomass growth (Fig 5A and 5B). To achieve similar biomass growth, conversion efficiency of *B*. *aeruginosa* should be 3 times higher than that of *Pl*. *corneus*, when the latter should be about 2 times higher that of *Po*. *canaliculata* (Fig 5A); the *per capita* biomass loss rate of *Pl*. *corneus* should be 5 times higher than that of *B*. *aeruginosa*, when the latter should be about 1.5 times higher than that of *Pl*. *corneus* (Fig 5B).

## Discussion

Using a comparative functional response experiment and simulations of the population dynamics of the snail consumers, we demonstrated that FRs could be predictive of both the invasiveness and ecological impacts of alien herbivorous snail species, providing a convenient way to predict the interactions between alien consumers and local resources, the population development of alien species and their potential ecological influence on native communities.

Further, although the comparative functional response approach in invasion ecology has thus far only been employed for analyzing predator-prey dynamics, we show with our present study that this could be extended to other taxonomic and trophic groups, such as herbivore-plant interactions [50,51]. Generally, there are three types of FR, with hyperbolic Type II FRs being generally de-stabilizing to prey/resource populations, and sigmoidal Type III FRs perhaps imparting stability to such population dynamics [52]. The Type II FR was thought to associate with specific consumer types such as invertebrates, while the Type III FR was thought to occur mainly in vertebrate consumers [3]. Switching between resources (e.g. two prey species) by generalist consumers with the declining density of those resources could contribute to Type III FRs. In addition, spatial refuge is another main cause underling the Type III FR [44]. To differentiate the types of FR for herbivorous snails, we used polynomial regression to analyze the first and second order terms [13]. In the present study, the Type II FR was found as the best description of all three herbivores feeding on the four plant species, while no evidence was found to suggest snails exhibited Type III FRs. This result was in accord with some recent research concerning comparative FRs that showed Type II FRs are the common Type for laboratory derived FRs [6,15,53]. It has been suggested that the Type of FR may not be fixed and is likely to change with the habitat characteristics, population development and body size [44,54]. In our experiment, simple habitat, a short experimental duration and similar body size likely made the expression of Type III FRs unlikely. In addition, some features of our experiment design might result in the occurrence of Type II FR [55]. That is, in our experiment, each snail was fed with one species of plant in a tank, thus resource selectivity (switching) and refuge did not exist, potentially contributing to the Type II FR. Nevertheless, the difference in magnitude of FR parameters between invaders and natives successfully predicts invasiveness and ecological impact, and the present herbivore-plant study is consistent with recent predator-prey studies [6,15–17,56].

Higher FR magnitudes for more invasive and ecologically damaging species may be due to differences in physical characteristics, such as resource acquisition and digestion ability, or the lack of co-evolutionary history between alien consumer and native resource [2]. When some other factors such as predator dependence [36], trait-mediated indirect interactions [37], intra-guild predation [57], body mass [58], temperature [59] and habitat heterogeneity [60], may impart influences on FR as well, our experimental design focused on the relationship between invasiveness/impact and simply-derived FR magnitude. Although the shape of FR did not alter with the invasiveness of species, the greater magnitude for the invasive species than the non-invasive and native species indicated that this process could be use as a tool to explore the invasive potential and ecological consequences of a range of alien species across taxonomic and trophic groups.

Including non-invasive alien species in the comparative FR framework enables us to address the relationship between invasiveness of alien species and FR, rather than only assessing ecological consequences of invasive species as recently advocated [3]. This distinction between invasiveness and impact is important, as they do not predict each other [4]. As demonstrated by van Kleunen et al. [1], to determine the traits and processes associated with invasiveness explicitly, comprehensive comparisons among invasive alien, non-invasive alien and native species are required. The comparison between invasive alien and native species could indicate whether invasive species will impact resource populations more so than natives, while comparing invasive and non-invasive alien species is probably the most direct test of determinants of invasiveness. Although the comparison between non-invasive alien and native species does not directly test for determinants of invasiveness, it might shed light on whether observed differences between invasive alien and native species reflect true determinants of invasiveness rather than general differences between alien and native species [1]. For example, some invasive plants and vertebrates with specific characteristics that make them attractive for ornamental or agricultural purposes were introduced tendentiously, but these characteristics may not play a role in invasiveness; thus, it should be combined with the comparison between non-invasive alien and native species to explicitly demonstrate the traits or processes associated with invasiveness. In our study, the invasive alien species, *Po*. *canaliculata*, had a much higher attack rate than the non-invasive alien, *Pl*. *corneus*, and the native species, *B*. *aeruginosa*, while this feeding parameter was also significantly different between non-invasive alien and native species. This indicates explicitly that the invasive, ecologically damaging species was associated with a high attack rate, and that the non-invasive alien species probably has some potential to impact on the native community. Given that attack rates represent the initial slope (a measure of relative consumption rate at low resource density), and as this can impart instability on resource populations [3], high attack rates intuitively can predict invasiveness and ecological impact. For the handling time and estimated maximum feeding rate of the snail consumers, the result was similar. The invasive alien species had shorter handling times and greater estimated maximum feeding rates than the native species, with both parameters of the non-invasive alien species intermediate between the two. Collectively, our results indicate that there is likely to be a continuum in the degree of invasiveness and ecological impact that correlates with FR parameters.

Our simulations further enhance our confidence in the use of this method as an effective tool in predicting invasiveness and impact of alien species, as we explicitly modelled the population level consequences of FRs for the consumer (i.e. numerical response). The invasive alien species with a high FR resulted in rapid population growth for the consumer itself. This explicitly indicated that high FR could convert into high invasive potential. In our simulation of the herbivorous snail populations, although one study has shown that the conversion efficiency of native and alien snails was not different [34], we varied this parameter given that the alien snails in the novel environment may have lower conversion effciencies than the natives. We found that the native snails needs about 6 times higher conversion efficiency than the invaders in order to achieve similar biomass growth (i.e. numerical response of the snail populations). We also examined the possible effect of consumer density dependence. We found that the invasive species would need to suffer about 7.5 times higher *per capita* biomass loss rate than the natives in order to achieve similer biomass growth for both species. Further, according to field dynamics records, for the native species, *Bellamya aeruginosa*, a female snail can give birth to about 50 individuals per generation [31,61], whereas for the invasive species, *Pomacea canaliculata*, about 700 individuals per generation are born [62]. This concurs with our results of simulations of the population dynamics of these species. Overall, since the invasive species has greater FRs (i.e. *per capita* effects) and a greater numerical response, these two features combined will result in a higher overall impact on plant populations, known as the “total response” (see [3]) or invader “impact” [63]. Since higher FRs are also apparently associated with higher numerical responses, then the easier direct measure, that is laboratory derived FRs, appear to be a rapid and reliable indicator of the likely invasiveness and ecological impact of alien species.

In our study, two herbivorous invertebrate species with different invasiveness and ecological impact, and one native counterpart, were compared with respect to experimentally derived functional responses and, further, we explored simulations of population effects for the consumer. On the basis of this, we conclude a likely correlation of invasiveness and ecological impact with FRs, but the limited range of species in the present study cannot yet fully test this hypothesis. However, our study adds to a growing number of others that show comparative FRs to be a highly effective way to both understand and predict invaders and their impacts [3]. In the future, through further dividing the invasive and non-invasive alien species into subcategories, such as introduced alien species not occuring outside of cultivation, species occasionally escaping but failing to establish in the field, species forming self-sustaining populations and invasive species spreading and causing varying degrees of damage [1], we could explore the relationship between FRs and invasiveness and impact more clearly. Further, by incorporating environmental variables into model building and experimental design with respect to FRs, we may predict invasive ability and potential impact against the background of global environmental change.

## Supporting Information

S1 Fig Relationships between initial plant biomass and proportion of plant consumed. (DOC)

S1 Table Parameter estimates from polynomial regression analyses of proportion of plant biomass consumed against initial plant biomass for the three snail species toward the four plant species. (DOC)
