# Supplementary material for: Comparative Functional Responses Predict the Invasiveness and Ecological Impacts of Alien Herbivorous Snails
Source: PLoS One. 2016 Jan 15;11(1):e0147017. doi: 10.1371/journal.pone.0147017 (PMC4714930; doi:10.1371/journal.pone.0147017)
Supplement: S1 Fig — (DOC) [file pone.0147017.s001.doc]

**Fig S1.** The proportion of plant biomass consumed of (A) *Ipomoea aquatica*, (B) *Cabomba caroliniana*, (C) *Hygrophila difformis* and (D) *Rotala indica* when fed on by the invasive alien species *Pomacea canaliculata*, and of the same plant species when fed on by the non-invasive alien species *Planorbarius corneus* (E, F, G, H) and the native species *Bellamya aeruginosa* (I, J, K, L); modelled by polynomial regression; *n* = 3 per initial plant biomass.
