# Supplementary material for: Comparative Functional Responses Predict the Invasiveness and Ecological Impacts of Alien Herbivorous Snails
Source: PLoS One. 2016 Jan 15;11(1):e0147017. doi: 10.1371/journal.pone.0147017 (PMC4714930; doi:10.1371/journal.pone.0147017)
Supplement: S1 Table — (DOC) [file pone.0147017.s002.doc]

**Table S1. Parameter estimates from polynomial regression analyses of proportion of plant biomass consumed against initial plant biomass for the three snail species (*Pomacea canaliculata*, *Planorbarius corneus and Bellamya aeruginosa*) toward the four plant species (*Ipomoea aquatica*, *Cabomba caroliniana*, *Hygrophila difformis* and *Rotala indica*). Values for the first and second order are present with *P* values. Significant *P* values are in bold, *α* = 0.05. Estimates for each snail species-plant species are presented followed by each snail species-pooled plant species.**

| Species of snails/plants | First order (*P* value) | Second order (*P* value) | Functional response type |
| --- | --- | --- | --- |
| *Pomacea-Ipomoea* | -0.737 (**< 0.001**) | 0.251 (**0.022**) | II |
| *Pomacea-Cabomba* | -0.627 (**0.003**) | 0.269 (**0.049**) | II |
| *Pomacea-Hygrophila* | -0.249 (**0.004**) | 0.047 (0.314) | II |
| *Pomacea-Rotala* | -0.571 (**0.003**) | 0.275 (**0.035**) | II |
| *Planorbarius-Ipomoea* | -0.330 (**0.041**) | 0.053 (0.657) | II |
| *Planorbarius-Cabomba* | -0.262 (**0.025**) | 0.098 (0.261) | II |
| *Planorbarius-Hygrophila* | -0.107 (**0.068**) | 0.057 (0.261) | II |
| *Planorbarius-Rotala* | -0.154 (**0.040**) | 0.095 (0.139) | II |
| *Bellamya-Ipomoea* | -0.173 (**< 0.001**) | 0.038 (**0.003**) | II |
| *Bellamya-Cabomba* | -0.100 (**0.034**) | 0.044 (0.233) | II |
| *Bellamya-Hygrophila* | -0.107 (**0.001**) | 0.061 (**0.009**) | II |
| *Bellamya-Rotala* | -0.107 (0.197) | 0.053 (0.485) | II |
| *Pomacea-pooled* | -0.546 (**< 0.001**) | 0.210 (**0.017**) | II |
| *Planorbarius-pooled* | -0.213 (**0.001**) | 0.076 (0.051) | II |
| *Bellamya-pooled* | -0.122 (**0.007**) | 0.049 (0.109) | II |
